# Levels and potential drivers of under‐five mortality sex ratios in low‐ and middle‐income countries

**DOI:** 10.1111/ppe.12763

**Published:** 2021-05-26

**Authors:** Janaína Calu Costa, Ann M. Weber, Safa Abdalla, Gary L. Darmstadt, Cesar G. Victora

**Affiliations:** ^1^ International Center for Equity in Health Postgraduate Program in Epidemiology Federal University of Pelotas Pelotas Brazil; ^2^ School of Community Health Sciences University of Nevada Reno NV USA; ^3^ Department of Pediatrics Stanford University School of Medicine Stanford CA USA

**Keywords:** child health, child mortality, developing countries, models, sex ratio, statistical, surveys and questionnaires

## Abstract

**Background:**

Non‐biological childhood mortality sex ratios may reflect community sex preferences and gender discrimination in health care.

**Objective:**

We assessed the association between contextual factors and gender bias in under‐five mortality rates (U5MR) in low‐ and middle‐income countries.

**Methods:**

Full birth histories available from Demographic and Health Surveys and Multiple Indicator Cluster Surveys (2010‐2018) in 80 countries were used to estimate U5MR male‐to‐female sex ratios. Expected sex ratios and their residuals (difference of observed and expected) were derived from a linear regression model, adjusted for overall mortality. Negative residuals indicate more likelihood of discrimination against girls, and we refer to this as a measure of potential gender bias. Associations between residuals and national development and gender inequality indices and with survey‐derived child health care indicators were tested using Spearman's correlation.

**Results:**

Mortality residuals for under‐five mortality were not associated with national development, education, religion, or gender inequality indices. Negative residuals were more common in countries where boys were more likely to be taken to health services than girls (rho −0.24, 95% confidence interval −0.45, −0.01).

**Conclusions:**

Countries where girls were more likely to die than boys, accounting for overall mortality levels, were also countries where boys were more likely to receive health care than girls. Further research is needed to understand which national characteristics explain the presence of gender bias, given that the analyses of development levels and gender equality did not discriminate between countries with or without excess mortality of girls. Reporting on child mortality separately by sex is required to enable such advances.

**Synopsis**

**Study question**

Which national characteristics—including indicators of levels of development, gender inequality, or gender bias in childcare—can predict male‐to‐female sex ratios in under‐five mortality, after adjusting for total morality level?

**What’s already known**

Sex ratios in under‐five mortality rates vary around the world, according to overall levels and causes of mortality, and to the presence of gender bias. Contextual factors associated with sex differentials in mortality have been explored, but so far studies failed to take overall mortality levels into account.

**What this study adds**

After adjustment for total mortality, higher‐than‐expected female mortality was found in countries where there was also evidence of gender bias in care‐seeking for common illnesses.

## 1 BACKGROUND

Sex differences in mortality are observed along the life cycle in association with various biological and social factors.^1^, ^2^ Overall mortality of children under 5 years of age is an important indicator of population development, whereas the unexpected differences in mortality sex ratios may reflect community sex preference and gender discrimination in child health care.^3^, ^4^

Under circumstances where boys and girls are equally valued and health care is evenly available to both sexes, boys have higher mortality rates than girls due to biological differences.^1^, ^2^, ^5^ The expected sex ratio in under‐five mortality rates (U5MR), however, varies geographically according to the causes and overall levels of mortality.^6^, ^7^

The survival advantage of girls becomes more evident as overall mortality falls because perinatal causes and malformations, which represent the leading causes of death when mortality is low, predominate among male infants.^7^, ^8^, ^9^ Nonetheless, in some countries and regions, girls are dying more often than expected and present similar or even higher mortality rates as their male counterparts, thus lowering the observed sex ratios.^7^ Where sex ratios in mortality deviate from the expected due to higher female mortality, gender bias must be considered as an underlying cause.

Documentation of sex ratios according to mortality levels can be used to identify where potential gender bias in childhood mortality exists. Over the past decades, different approaches and methods have been used for assessing the presence of excess female deaths based on overall or male mortality rates.^6^, ^10^, ^11^, ^12^, ^13^ Some studies have used descriptive approaches to identify LMICs with sex ratios greater or lower than 1.0,^13^ or used mathematical models to identify outliers using data from all high, middle, and low‐income countries with available information.10 We refer to these as descriptive, because all countries with information were included in the models, without excluding those with likely gender bias in child health care or other societal aspects. In contrast, prescriptive approaches have been proposed, starting with the selection of countries where gender discrimination or preference is assumed to be absent, and using these data to derive expected mortality levels by sex or sex ratios according to male mortality.^6^, ^11^ Prescriptive models, as would be expected, tend to identify more countries presenting gender bias than purely descriptive approaches, although both methods result in similar rankings of countries in terms of excess mortality among girls.^14^

Some authors explored multiple contextual factors associated with sex differentials in childhood mortality, including socio‐economic development level and measures of gender discrimination, but failed to take the overall level of mortality into account in their analyses.^13^, ^15^

In this article, we address an issue that has not been previously investigated in the literature in multiple countries. After adjusting sex ratios by overall under‐five mortality rates, we assessed whether differences between observed and expected sex ratios may be predicted by national indices of development and gender inequalities, or by survey‐based measures of gender bias in child health care.

## 2 METHODS

### 2.1 Data sources

We analysed data from nationally representative Demographic and Health Surveys (DHS) and Multiple Indicator Cluster Surveys (MICS) carried out in LMICs from 2010 onwards. For countries with more than one survey, we selected the most recent. These surveys collect comparable data across countries based on standard questionnaires. We aimed to include all surveys with public‐domain data sets available on the DHS and MICS websites (http://dhsprogram.com and http://mics.unicef.org, respectively), which had information on full birth histories for women of reproductive age.

### 2.2 Outcome

The probability of a child dying between birth and the fifth birthday, known as U5MR, was estimated from full retrospective birth history using standard DHS methods.^16^ Women aged 15 to 49 years were asked about pregnancies and deliveries in the years preceding the survey, including characteristics such as birth date, sex of the child, and survival status. If a child was not alive, the age at death was recorded. From these questions, it was possible to calculate U5MR using a synthetic cohort life table approach in which probabilities of survival for small age segments were combined to calculate the probability of surviving up to age five (the mortality rate being calculated as 1 minus the probability of survival).^16^, ^17^ Estimates were based on the 10 years preceding the survey to ensure a reasonable sample size after stratification. Mortality rates are presented as the number of deaths per 1000 live births. From the rates disaggregated by sex, male‐to‐female ratios were estimated. In order to facilitate the interpretation, sex ratios are presented multiplied by 100.

### 2.3 Covariates

For contextual factors that could affect mortality differentials by sex, we used indices of development and gender inequality at the national level, available from external databases.

Based on studies from Tabutin and Willems^13^ and Fuse and Crenshaw,^15^ we selected similar development indices to assess associations with sex ratio residuals. From the World Bank repository, we included gross domestic product (GDP) per capita, based on purchasing power parity (constant international dollar),^18^ annual rate of growth of GDP,^19^ total health expenditure per capita as a percentage of GDP,^20^ percentage of the total economically active population employed in industry^21^ and services,^22^ adult literacy rate of men and women aged 15 and above (those who can read and write with understanding a short statement about everyday life),^23^ difference between boys^24^ and girls^25^ in primary school enrolment, mean years of schooling for women as a percentage of those for men,^24^, ^25^ women's secondary education enrolment rate,^26^ life expectancy at birth,^27^ and total fertility rate for women aged 15 to 49 years.^28^

We obtained information from the Pew Forum on Religion and Public Life repository on percentages of Christian and Muslim population by country.^29^ The study by Fuse and Crenshaw^15^ also included the percentage of Buddhists in each country, but in our analyses, there were only six countries where this proportion was above 1%, and we opted to leave out this variable. In addition, we acquired data from the United Nations Development Programme (UNDP) on women's average age at first marriage as well as the Human Development Index, a summary measure of average achievements in life expectancy, education, and standard of living.^15^, ^30^

Gender inequality indices are composite measures that combine data on factors such as health, economics, politics, empowerment, and labour participation. Four gender indices were chosen: Gender Development Index (GDI) and Gender Inequality Index (GII), from UNDP^30^; Social Institutions and Gender Index (SIGI), from the Organization for Economic Co‐operation and Development^31^; and Women, Peace and Security Index (WPS), from the Georgetown Institute for Women, Peace and Security.^32^ For GDI, GII, and SIGI, higher absolute values represent greater gender inequalities in each country, whereas for WPS the reverse is true. Definitions and data sources are detailed in Supplementary Table S1.

Because information on contextual variables is not available for all calendar years, we used data for the closest year to the mid‐point of the 10‐year interval over which births were studied in each survey, that is 5 years before the survey date.

The assessment of factors potentially associated with gender bias in U5MR was complemented by analyses using child health care indicators available from the same surveys used for mortality estimates, in order to investigate whether the sex ratio in mortality could be related to the sex ratio in health care utilization. The following indicators were analysed: (a) care‐seeking for common childhood symptoms: proportion of children under 5 years of age who presented with symptoms of pneumonia (short, rapid breathing which was chest‐related and/or difficult breathing), fever, or diarrhoea in the 2 weeks prior to the interview and were taken to an appropriate health care provider (the definitions of appropriate providers are country‐specific, but neither pharmacists, drug sellers, or traditional practitioners were included); (b) full immunization coverage: proportion of children aged 12 to 23 months who received at any age all the following vaccines: one dose of BCG (Bacille Calmette‐Guérin), three doses of DPT (diphtheria, pertussis, and tetanus) or tetravalent/pentavalent vaccine, three doses of polio, and at least one dose of measles (either as monovalent vaccine or as measles‐containing vaccine combinations with other immunogens); and (c) postnatal care for babies: proportion of last‐born children under 2 years of age who received any health check‐up within two days after birth.

### 2.4 Statistical analysis

Linear regression was used to model the association between U5MR sex ratio and total U5MR (for both sexes combined). The analyses included all countries for which mortality data were available. Sex ratio residuals were estimated for each country as the difference between observed and predicted values from the regression model. We refer to residuals as a measure of potential gender bias, as positive values indicate higher mortality than expected among boys than girls, which we interpret as a lower likelihood of discrimination against girls. Negative residuals signal higher‐than‐expected female mortality and thus indicate the presence of potential gender bias disadvantaging girls.

Next, we tested the association of covariates with the above‐mentioned residuals, using Spearman's correlation coefficient due to the non‐normal distribution of the variables.

### 2.5 Sensitivity analyses

Sensitivity analysis is presented in the supplementary materials and includes estimates of sex ratio residuals for countries according to the methods proposed by Alkema et al.^10^ and Guilmoto et al.^11^ The former is a descriptive model and defines sex ratio higher or lower than expected based on the modelled global relations between mortality levels and sex ratios.^10^ The latter used expected female U5MR based on the relation between male and female U5MRs observed in 46 countries without known gender discrimination in different periods.^11^

Additional sensitivity analyses included repeating the main correlation analysis for a subset of 52 countries with information on all explanatory variables with 70 or more data points, thus excluding four of the original set of 22 variables.

All analyses were carried out in Stata 15 (StataCorp, College Station, TX, USA). The agencies responsible for the surveys provide in each data set the sampling weights, based on selection probability, and adjusted for non‐response, which ensure the representativeness of the sample and are used to correct for the under‐ or over‐sampling of different strata during sample selection. The country‐ and sex‐specific mortality rates estimated from these data sets account for the complex survey design, including sampling weights and the clustered nature of the data. More information about the estimation and use of sampling weights is available elsewhere.^16^

### 2.6 Ethics approval

Ethical clearance was obtained by the national agencies responsible for each survey and all identifying data are omitted guaranteeing participants’ anonymity.

## 3 RESULTS

A total of 82 countries had information on mortality rates by sex. We removed two countries identified as outliers from the analyses for U5MR (Albania and Armenia); the results for the complete data set including these two countries are presented in the Supplementary Materials (Figure S1) and the surveys included in the analyses are listed in the Supplementary Table S2. Table 1 shows that the mean U5MR in the 80 countries was 58 per thousand live births [standard deviation (SD) 33], ranging from 16 in Moldova to 153 in Niger. Summary statistics for the sex ratios are also presented in Table 1.

**TABLE 1:** Descriptive statistics of under 5 mortality rates (deaths per 1000 live births) from 80 countries

	Mean	Std. Dev.	Median	P25	P75	Minimum	Maximum
Total	58	33	54	30	78	16	153
Male	63	36	57	30	85	16	160
Female	53	32	51	27	68	14	146
Male‐to‐female sex ratio	119	13	119	110	125	78	156

**FIGURE 1:**
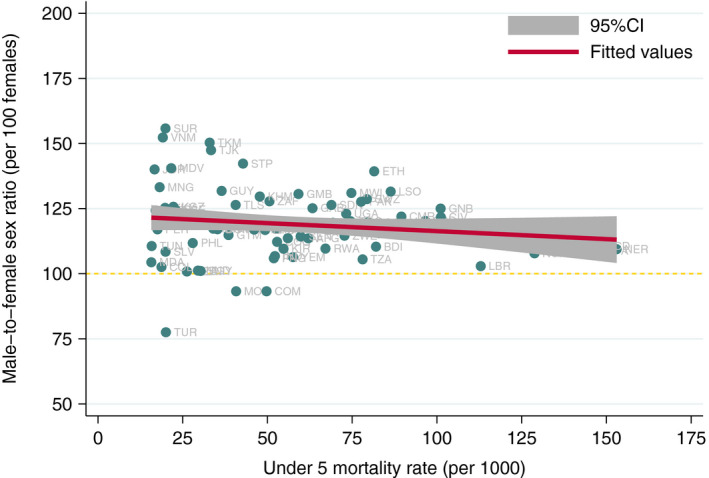
Country‐specific male‐to‐female sex ratios by total under 5 mortality rate. Note: Horizontal dashed line represents the equal mortality rates for boys and girls

Figure 1 shows the scatter of the observations—sex ratios and total mortality level—as well as the fitted line corresponding to the model. We observe higher male‐to‐female sex ratios when overall mortality rates are low, which decreases as total U5MR increases, reaching values close to 100.

Potential gender bias against girls is observed when residuals are negative, reflecting lower‐than‐expected sex ratios after adjustment for total U5MR, that is fewer deaths of boys and more deaths of girls than would be expected. Residuals of U5MR sex ratios ranged from −43.7 to 34.5 (mean 0, SD 13.3, and median 0.11). Skewness and kurtosis test for normality showed a *P*‐value of .124, presenting no evidence that residuals were not normally distributed. Supplementary Figure S2 shows the distribution of the residuals.

The rank correlation coefficients between the residuals with explanatory variables at national level are presented in Table 2. We found no evidence of the association between sex ratio residuals for U5MR and any national indicators including GDP, Human Development Index, religion, age at first marriage, fertility, employment, education, or gender inequality indices.

**TABLE 2:** Spearman's correlation coefficient between under 5 mortality sex ratio residuals and explanatory variables

Group	Development and gender indices	N^a^	rho (95% CI)
Economics	Gross domestic product (GDP), per capita	80	0.05 (−0.17, 0.27)
	Gross domestic product, annual growth	79	0.09 (−0.14, 0.30)
	Health expenditure (% of GDP)	77	−0.02 (−0.24, 0.21)
Employment	Labour force in industry	78	0.14 (−0.08, 0.35)
	Labour force in services	78	0.11 (−0.12, 0.32)
Education	Adult literacy rate	74	0.12 (−0.11, 0.34)
	Primary education (difference between boys and girls)	75	−0.02 (−0.25, 0.21)
	Female secondary enrolment	72	0.04 (−0.19, 0.27)
	Female mean years of schooling (% of male)	74	0.17 (−0.06, 0.38)
Demographics	Life expectancy	80	−0.05 (−0.27, 0.17)
	Female average age at first marriage	53	0.26 (−0.01, 0.50)
	Total fertility rate	80	−0.01 (−0.23, 0.21)
	Human Development Index	77	0.04 (−0.19, 0.26)
Religion	Christian	79	−0.07 (−0.29, 0.15)
	Muslim	79	−0.01 (−0.23, 0.21)
Gender	Gender Development Index	73	0.04 (−0.19, 0.27)
	Gender Inequality Index	67	−0.13 (−0.36, 0.12)
	Social Institutions and Gender Index	67	0.07 (−0.17, 0.31)
	Women, Peace and Security index	70	0.07 (−0.16, 0.30)
Child health (male‐to‐female sex ratios)	Postnatal care for babies	56	0.01 (−0.25, 0.27)
	Full immunization coverage	76	0.11 (−0.12, 0.32)
	Care‐seeking for common illnesses	71	−0.24 (−0.45, −0.01)

Note

Negative values for residuals indicate more likelihood of gender bias against girls.

^a^ Sample sizes vary according to data availability.

**FIGURE 2:**
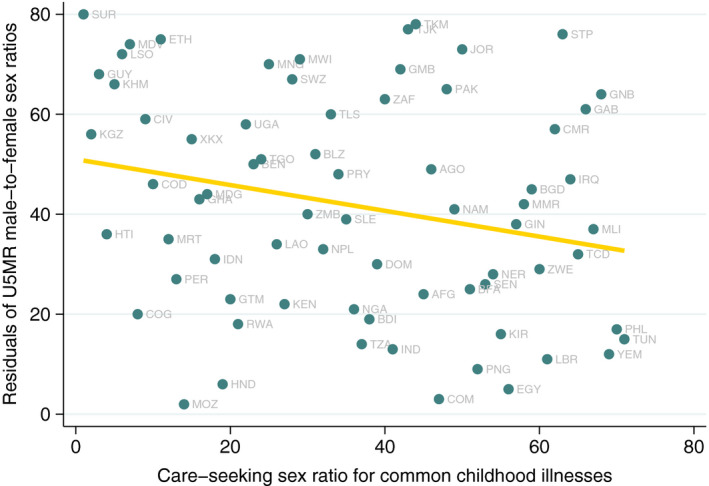
Rankings of male‐to‐female under 5 mortality residuals and sex ratio of care‐seeking for common childhood illnesses

Next, we tested associations between the residuals and sex ratios for three child health care indicators derived from the same surveys. There was no evidence of associations with sex ratios in postnatal care or full immunization coverage. However, the male‐to‐female ratio in care‐seeking for common diseases [rho −0.24, 95% confidence interval (95% CI) −0.45, −0.01] was associated with mortality residuals in the expected direction (Figure 2).

## 4 COMMENT

### 4.1 Principal findings

Our findings confirm prior research showing that the male‐to‐female sex ratio in mortality is inversely associated with overall U5MR level.^6^, ^7^, ^10^ The analyses of correlations between mortality sex ratio residuals and national characteristics failed to show associations with national wealth, women's characteristics, or gender inequality indices. The only explanatory variable with statistical evidence of an association with the residuals was the sex ratio for care‐seeking for common illnesses: countries where boys were more likely than girls to receive care when sick were also those with evidence of mortality bias against girls. Although the 95% confidence intervals included the null value, the correlation coefficients with mortality residuals were in the expected direction for female mean years of schooling (as a percentage of male schooling) and female mean age at first marriage.

### 4.2 Strengths of the study

We presented a novel approach by quantifying potential gender bias through residuals of the sex ratio for mortality, adjusted for overall mortality level. When residuals are positive, the mortality of boys is higher than would be expected based on the mortality of girls, thus suggesting that gender bias against girls is unlikely. Our approach incorporates variability in sex ratios due to causes of death and adjusts for the biological female advantage in survival. Instead of using a fixed value for the expected sex ratio, we were able to model such ratios according to the overall mortality level, leading to estimates of expected sex ratios for a set of LMICs and allowing us to calculate residuals.

### 4.3 Limitations of the data

Some limitations ought to be recognized. Estimation of mortality rates for children is based upon maternal recall of births and deaths obtained in household sample surveys and may be affected by recall bias, misdating errors, and omission.^16^ In general, the interpretation of a lower‐than‐expected mortality sex ratio is that there is an excess of female deaths.^10^ However, in some populations, the interpretation might be that some boys who died were omitted.^33^ Also, higher‐than‐expected mortality sex ratios could reflect the omission of the deaths of girls. Nevertheless, a review of this issue found little or inconclusive evidence of systematic sex‐specific omission of births ^33^, ^34^, ^35^ or deaths.^6^

It is also interesting to highlight the case of Armenia, found to be an outlier in the distribution of sex ratios by overall mortality. The country presents a low fertility rate, low mortality level, and documented discrimination against girls.^36^, ^37^ These estimates, however, must be taken into account with caution, due to the small number of numbers and deaths.^38^

Additionally, some degree of survival bias may exist when assessing coverage with child health interventions, because these questions are asked for children who are still alive, and gender bias in mortality may have already happened. Childhood mortality sex ratios may also be affected by sex‐selective abortions, which would restrict the number of unwanted births of girls, and ensure higher survival among those girls who are born.^15^, ^39^ It is important to point out that some within‐country variations in mortality levels as well as in gender bias in mortality exist, as already reported for some countries, such as India (where mortality is higher and gender bias against girls is more likely to happen in the Northern regions).^11^ In addition, single surveys can be subject to random error to a greater extent than in the case for mortality estimates based upon multiple surveys over time from the same country.^40^ Lastly, we found evidence in one pairwise association out of 22 comparisons, so that chance cannot be ruled out; nevertheless, we note that the association between possible gender bias in mortality and health care was in the expected direction.

### 4.4 Interpretation

Our exploratory analysis and selection of possible drivers of gender bias in mortality were based on two previous studies. Using data from LMICs in the 1980s, Tabutin and Willems^13^ address sex ratio of mortality at ages 1‐4 years (child mortality rate, CMR) and found no relationship with the level of social and educational development attained by countries and regions. In these analyses, GDP and Human Development Index presented weak correlations with CMR sex ratios.^13^ A weak association was found in the bivariate analyses performed by Fuse and Crenshaw^15^ of sex differentials in infant mortality rate (IMR, 0‐1 year of age) for all countries with available information, including high‐income geographies. In this latter study, evidence of an association between IMR sex ratios and national income was found after adjustment for several covariates.^15^

Regarding the education indicator, the direction of the association agrees with the literature. Results from Sub‐Saharan Africa and South Asia indicate that as maternal education increases, so does the male‐to‐female mortality ratio.^34^ In another set of LMICs, indicators of inequality in education between men and women were associated with excess female deaths in the expected direction.^13^ The above‐mentioned global study found that female secondary education enrolment was associated with sex ratios for IMR in the expected direction.^15^ Education levels reflect the social position of women, which is supported by other research showing the positive impact of female education in decreasing overall child mortality.^41^

Another indicator that might reflect women's position in society is the mean age at first marriage. In our analyses, we found that countries with an older average age at marriage may show less evidence of gender bias against girls in mortality. A correlation in the same direction had been reported previously, by Tabutin and Willems.^13^ Another global study of the association between sex differentials in U5MR and the Gender Inequality Index found that in more unequal societies girls face lower survival chances relative to boys, especially in low‐ and middle‐income countries.^42^

Our finding of higher‐than‐expected mortality residuals may be interpreted as representing either neglect of girls, or improved care for boys. The correlation with gender‐specific care‐seeking suggests that higher rates for boys account, at least in part, for the observed mortality differences. These findings are also corroborated by our previous analysis of gender bias in care‐seeking practices in 60 DHS,^43^ which was also associated with excess female mortality using the external references proposed in the earlier literature.^10^, ^14^, ^44^ Gender‐based discrimination in child health care has been reported in specific settings, mostly in South Asia and China.^4^ It has been argued that excess female mortality among children has a multidimensional nature and is more likely to happen if there is sex discrimination in nutrition or health care, but that this also depends on the cause‐of‐death pattern observed in each population.^8^

In our sensitivity analyses, we calculated residuals using the equations proposed in previous studies for estimating sex differences in under‐five mortality.^10^, ^11^ These approaches produced similar direction of associations for most of the explanatory variables (Supplementary Table S3). We also repeated the correlation analyses for 52 countries with information on 18 explanatory variables, and results remained virtually unchanged (Supplementary Table S4).

## 5 CONCLUSIONS

In conclusion, our results confirm the importance of reporting on child mortality separately by sex and contribute to the existing literature by updating previous research on the association of mortality sex ratios and contextual factors. Contrary to our original set of hypotheses, national characteristics were not correlated with mortality residuals, except for higher care‐seeking for boys than for girls.

Further research is needed to refine the analyses of explanatory factors for gender‐specific mortality.

## Supporting information

Supplementary Material

## Data Availability

The data that support the findings of this study are available in DHS and MICS websites (http://dhsprogram.com and http://mics.unicef.org, respectively).
