# Supplementary material for: Levels and potential drivers of under‐five mortality sex ratios in low‐ and middle‐income countries
Source: Paediatr Perinat Epidemiol. 2021 May 26;35(5):549–56. doi: 10.1111/ppe.12763 (PMC8453971; doi:10.1111/ppe.12763)
Supplement: Supplementary file 1 — Supplementary Material [file PPE-35-549-s001.docx]

# Levels and potential drivers of under-five mortality sex ratios in low- and middle-income countries - Supplementary Materials

**eTable 1 – Definition and data source of potential drivers of sex differentials in U5MR.**

| **Variable** | **Description** | **Source** |
| --- | --- | --- |
| Gross Domestic Product (GDP) *per capita* | “GDP is gross domestic product converted to international dollars using purchasing power parity rates (PPP). GDP at purchaser's prices is the sum of gross value added by all resident producers in the economy plus any product taxes and minus any subsidies not included in the value of the products. It is calculated without making deductions for depreciation of fabricated assets or for depletion and degradation of natural resources. An international dollar has the same purchasing power over GDP as the U.S. dollar has in the United States. Data are in current international dollars based on the 2011 International Comparison Program (ICP) round.” | World Bank |
| Rate of growth of GDP (annual %) | “Annual percentage growth rate of GDP at market prices based on constant local currency. Aggregates are based on constant 2010 U.S. dollars.” | World Bank |
| Current health expenditure (% of GDP) | “Level of current health expenditure expressed as a percentage of GDP. Estimates of current health expenditures include healthcare goods and services consumed during each year. This indicator does not include capital health expenditures such as buildings, machinery, IT and stocks of vaccines for emergency or outbreaks.” | World Bank |
| Labour force structure | Percentage of the total economically active population employed in industry. “Employment is defined as persons of working age who were engaged in any activity to produce goods or provide services for pay or profit, whether at work during the reference period or not at work due to temporary absence from a job, or to working-time arrangement. The industry sector consists of mining and quarrying, manufacturing, construction, and public utilities (electricity, gas, and water)” | World Bank |
| Labour force structure | Percentage of the total economically active population employed in services. “The services sector consists of wholesale and retail trade and restaurants and hotels; transport, storage, and communications; financing, insurance, real estate, and business services; and community, social, and personal services” | World Bank |
| Adult literacy rate | “Adult literacy rate is the percentage of people ages 15 and above who can both read and write with understanding a short simple statement about their everyday life.” | World Bank |
| Primary school enrolment among girls | Difference between boys and girls in primary enrolment. “Primary education provides children with basic reading, writing, and mathematics skills along with an elementary understanding of such subjects as history, geography, natural science, social science, art, and music.” | World Bank |
| Women’s educational status | Percentage of all women enrolled in secondary education. “Secondary education completes the provision of basic education that began at the primary level and aims at laying the foundations for lifelong learning and human development, by offering more subject- or skill-oriented instruction using more specialized teachers.” | World Bank |
| Mean years of schooling for women | “Average number of years of education received by people ages 25 and older, converted from educational attainment levels using official durations of each level”. We used the indicator as female mean years of schooling, as percentage that for men. | UNDP |
| Life expectancy at birth | “Life expectancy at birth indicates the number of years a new born infant would live if prevailing patterns of mortality at the time of its birth were to stay the same throughout its life.” | World Bank |
| Women's average age at first marriage | “Mean age at marriage shows the average length of single life expressed in years among those females who marry before age 50. It is a synthetic indicator calculated from marital status categories of men and women aged 15 to 54 at the census or survey date.” | World Bank |
| Total fertility rate | “Represents the number of children that would be born to a woman if she were to live to the end of her childbearing years and bear children in accordance with age-specific fertility rates of the specified year.” | World Bank |
| Human Development Index | The Human Development Index (HDI) is a summary measure of average achievement in key dimensions of human development: a long and healthy life, being knowledgeable and have a decent standard of living. The HDI is the geometric mean of normalized indices for each of the three dimensions.  The health dimension is assessed by life expectancy at birth, the education dimension is measured by mean of years of schooling for adults aged 25 years and more and expected years of schooling for children of school entering age. The standard of living dimension is measured by gross national income per capita. The HDI uses the logarithm of income, to reflect the diminishing importance of income with increasing GNI. The scores for the three HDI dimension indices are then aggregated into a composite index using geometric mean. | UNDP |
| Christian | Percentage of Christian population | Pew Research Center |
| Muslim | Percentage of Muslim population | Pew Research Center |
| Gender Development Index | “GDI measures gender gaps in human development achievements by accounting for disparities in three basic dimensions: health, knowledge and living standards using the same component indicators as in the Human Development Index (HDI). The GDI is the ratio of the HDIs calculated separately for females and males using the same methodology as in the HDI. It is a direct measure of gender gap showing the female HDI as a percentage of the male HDI. Higher values of GDI represent greater deviation from parity in each country and takes equally into consideration gender gaps favouring males, as well as those favouring females.” | United Nations Development Programme |
| Gender Inequality Index | “A composite measure reflecting inequality in achievements between women and men in three dimensions: reproductive health, empowerment and the labour market.” GII with higher values represents greater gender inequality in each country. | United Nations Development Programme |
| Social Institutions and Gender Index | “The SIGI covers four dimensions of discriminatory social institutions, spanning major socio-economic areas that affect women’s lives: discrimination in the family, restricted physical integrity, restricted access to productive and financial resources and restricted civil liberties. The SIGI’s variables quantify discriminatory social institutions such as unequal inheritance rights, child marriage, violence against women, and unequal land and property rights. Higher SIGI values indicate higher inequality: from 0% for no discrimination to 100% for very high discrimination.” | Organization for Economic Co-operation and Development |
| Women, Peace and Security Index | “The Women, Peace, and Security Index is a comprehensive measure of women’s wellbeing spanning three dimensions: inclusion (economic, social, political); justice (formal laws and informal discrimination); and security (at the family, community, and societal levels)” | Georgetown Institute for Women, Peace and Security |

**eTable 2 – List of surveys included in the analyses**

| **Country (year)** | **source** |
| --- | --- |
| Afghanistan (2015) | DHS |
| Algeria (2012) | MICS |
| Angola (2015) | DHS |
| Bangladesh (2019) | MICS |
| Belize (2015) | MICS |
| Benin (2017) | DHS |
| Burkina Faso (2010) | DHS |
| Burundi (2016) | DHS |
| Cambodia (2014) | DHS |
| Cameroon (2018) | DHS |
| Chad (2014) | DHS |
| Colombia (2015) | DHS |
| Comoros (2012) | DHS |
| Congo (Brazzaville) (2014) | MICS |
| Congo, Democratic Republic (2017) | MICS |
| Côte d’Ivoire (2016) | MICS |
| Dominican Republic (2014) | MICS |
| Egypt (2014) | DHS |
| El Salvador (2014) | MICS |
| Eswatini (2014) | MICS |
| Ethiopia (2016) | DHS |
| Gabon (2012) | DHS |
| Gambia (2018) | MICS |
| Ghana (2017) | MICS |
| Guatemala (2014) | DHS |
| Guinea (2018) | DHS |
| Guinea Bissau (2014) | MICS |
| Guyana (2014) | MICS |
| Haiti (2016) | DHS |
| Honduras (2011) | DHS |
| India (2015) | DHS |
| Indonesia (2017) | DHS |
| Iraq (2018) | MICS |
| Jordan (2017) | DHS |
| Kenya (2014) | DHS |
| Kiribati (2018) | MICS |
| Kosovo (2013) | MICS |
| Kyrgyzstan (2018) | MICS |
| Lao (2017) | MICS |
| Lesotho (2018) | MICS |
| Liberia (2013) | DHS |
| Madagascar (2018) | MICS |
| Malawi (2015) | DHS |
| Maldives (2016) | DHS |
| Mali (2018) | DHS |
| Mauritania (2015) | MICS |
| Moldova (2012) | MICS |
| Mongolia (2018) | MICS |
| Mozambique (2015) | DHS |
| Myanmar (2015) | DHS |
| Namibia (2013) | DHS |
| Nepal (2016) | DHS |
| Niger (2012) | DHS |
| Nigeria (2018) | DHS |
| Pakistan (2017) | DHS |
| Papua New Guinea (2016) | DHS |
| Paraguay (2016) | MICS |
| Peru (2018) | DHS |
| Philippines (2017) | DHS |
| Rwanda (2014) | DHS |
| Sao Tomé and Principe (2014) | MICS |
| Senegal (2017) | DHS |
| Sierra Leone (2017) | MICS |
| South Africa (2016) | DHS |
| South Sudan (2010) | MICS |
| State of Palestine (2014) | MICS |
| Sudan (2014) | MICS |
| Suriname (2018) | MICS |
| Tajikistan (2017) | DHS |
| Tanzania (2015) | DHS |
| Timor-Leste (2016) | DHS |
| Togo (2017) | MICS |
| Tunisia (2018) | MICS |
| Turkey (2013) | DHS |
| Turkmenistan (2015) | MICS |
| Uganda (2016) | DHS |
| Vietnam (2013) | MICS |
| Yemen (2013) | DHS |
| Zambia (2018) | DHS |
| Zimbabwe (2019) | MICS |

**eTable 3 – Sensitivity analysis of the association between mortality sex ratios and selected predictors using Spearman’s correlation.**

| **Group** | **Country development and gender indices** | **Alkema et al (2014)** | | | | **Guilmoto et al (2018)** | | | |
| --- | --- | --- | --- | --- | --- | --- | --- | --- | --- |
|  |  | **N** | **rho** | **LL** | **UL** | **N** | **rho** | **LL** | **UL** |
| Economics | GDP per capita | 80 | -0.08 | -0.29 | 0.15 | 80 | -0.04 | -0.26 | 0.18 |
|  | GDP annual growth | 79 | 0.08 | -0.15 | 0.29 | 79 | 0.09 | -0.13 | 0.31 |
|  | Health expenditure (% of GDP) | 77 | 0.01 | -0.21 | 0.23 | 77 | -0.01 | -0.23 | 0.21 |
| Employment | Labour force in industry | 78 | 0.03 | -0.19 | 0.25 | 78 | 0.07 | -0.15 | 0.29 |
|  | Labour force in services | 78 | 0.00 | -0.23 | 0.22 | 78 | 0.03 | -0.19 | 0.25 |
| Education | Adult literacy rate | 74 | 0.01 | -0.22 | 0.24 | 74 | 0.04 | -0.19 | 0.27 |
|  | Primary education (difference between boys and girls) | 75 | -0.08 | -0.30 | 0.15 | 75 | -0.06 | -0.29 | 0.16 |
|  | Female secondary enrolment | 72 | -0.09 | -0.32 | 0.14 | 72 | -0.06 | -0.28 | 0.18 |
|  | Female mean years of schooling (% of male) | 74 | 0.06 | -0.17 | 0.28 | 74 | 0.09 | -0.14 | 0.31 |
| Demographics | Life expectancy | 80 | -0.21 | -0.41 | 0.01 | 80 | -0.17 | -0.38 | 0.05 |
|  | Female average age at first marriage | 53 | 0.17 | -0.11 | 0.42 | 53 | 0.18 | -0.09 | 0.43 |
|  | Total fertility rate | 80 | 0.12 | -0.11 | 0.33 | 80 | 0.08 | -0.14 | 0.30 |
|  | Human Development Index | 77 | -0.12 | -0.33 | 0.11 | 77 | -0.08 | -0.30 | 0.15 |
| Religion | Christian (%) | 79 | -0.05 | -0.27 | 0.17 | 79 | -0.07 | -0.28 | 0.16 |
|  | Muslim (%) | 79 | -0.01 | -0.23 | 0.21 | 79 | 0.01 | -0.21 | 0.23 |
| Gender | Gender Development Index | 73 | -0.04 | -0.27 | 0.19 | 73 | -0.03 | -0.26 | 0.20 |
|  | Gender Inequality Index | 67 | 0.00 | -0.24 | 0.24 | 67 | -0.03 | -0.27 | 0.21 |
|  | Social Institutions and Gender Index | 67 | 0.17 | -0.08 | 0.39 | 67 | 0.15 | -0.10 | 0.38 |
|  | Women, Peace and Security index | 70 | -0.03 | -0.26 | 0.21 | 70 | -0.01 | -0.24 | 0.23 |
| Child health  (sex ratios) | Postnatal care for babies | 56 | 0.01 | -0.25 | 0.27 | 56 | 0.01 | -0.25 | 0.27 |
|  | Full immunisation coverage | 76 | 0.13 | -0.10 | 0.34 | 76 | 0.13 | -0.10 | 0.34 |
|  | Care-seeking for common illnesses | 71 | -0.20 | -0.41 | 0.04 | 71 | -0.21 | -0.42 | 0.03 |

**eTable 4 – Sensitivity analysis comparing the correlation coefficients for the whole sample of 80 counties and a subset of 52 countries with information on all explanatory variables with 70 or more data points.**

| **Group** | **Development and gender indices** | **N** | **rho (95% CI)** |  | **N** | **rho (95% CI)** |
| --- | --- | --- | --- | --- | --- | --- |
| Economics | Gross domestic product (GDP), per capita | 80 | 0.05 (-0.17, 0.27) |  | 52 | 0.06 (-0.21, 0.33) |
|  | Gross domestic product, annual growth | 79 | 0.09 (-0.14, 0.30) |  | 52 | 0.05 (-0.23, 0.32) |
|  | Health expenditure (% of GDP) | 77 | -0.02 (-0.24, 0.21) |  | 52 | 0.04 (-0.23, 0.31) |
|  |  |  |  |  |  |  |
| Employment | Labour force in industry | 78 | 0.14 (-0.08, 0.35) |  | 52 | 0.15 (-0.13, 0.40) |
|  | Labour force in services | 78 | 0.11 (-0.12, 0.32) |  | 52 | 0.09 (-0.19, 0.35) |
|  |  |  |  |  |  |  |
| Education | Adult literacy rate | 74 | 0.12 (-0.11, 0.34) |  | 52 | 0.23 (-0.04, 0.48) |
|  | Primary education (difference between boys and girls) | 75 | -0.02 (-0.25, 0.21) |  | 52 | -0.11 (-0.37, 0.17) |
|  | Female secondary enrolment | 72 | 0.04 (-0.19, 0.27) |  | 52 | 0.16 (-0.12, 0.41) |
|  | Female mean years of schooling (% of male) | 74 | 0.17 (-0.06, 0.38) |  | 52 | 0.12 (-0.16, 0.38) |
|  |  |  |  |  |  |  |
| Demographics | Life expectancy | 80 | -0.05 (-0.27, 0.17) |  | 52 | -0.05 (-0.32, 0.23) |
|  | Female average age at first marriage | 53 | 0.26 (-0.01, 0.50) |  | Excluded |  |
|  | Total fertility rate | 80 | -0.01 (-0.23, 0.21) |  | 52 | -0.13 (-0.39, 0.15) |
|  | Human Development Index | 77 | 0.04 (-0.19, 0.26) |  | 52 | 0.09 (-0.19, 0.36) |
|  |  |  |  |  |  |  |
| Religion | Christian | 79 | -0.07 (-0.29, 0.15) |  | 52 | -0.05 (-0.32, 0.23) |
|  | Muslim | 79 | -0.01 (-0.23, 0.21) |  | 52 | -0.02 (-0.29, 0.26) |
|  |  |  |  |  |  |  |
| Gender | Gender Development Index | 73 | 0.04 (-0.19, 0.27) |  | 52 | 0.00 (-0.27, 0.27) |
|  | Gender Inequality Index | 67 | -0.13 (-0.36, 0.12) |  | Excluded |  |
|  | Social Institutions and Gender Index | 67 | 0.07 (-0.17, 0.31) |  | Excluded |  |
|  | Women, Peace and Security index | 70 | 0.07 (-0.16, 0.30) |  | 52 | 0.08 (-0.20, 0.34) |
|  |  |  |  |  |  |  |
| Child health (male-to-female sex ratios) | Postnatal care for babies | 56 | 0.01 (-0.25, 0.27) |  | Excluded |  |
|  | Full immunization coverage | 76 | 0.11 (-0.12, 0.32) |  | 52 | 0.14 (-0.14, 0.40) |
|  | Care-seeking for common illnesses | 71 | -0.24 (-0.45, -0.01) |  | 52 | -0.31 (-0.54, -0.04) |

**eFigure 1 – Distribution of U5MR sex ratios (times 100) by total U5MR and respective fractional polynomials fitted line for the full sample of countries (n = 82) and excluding Albania (the top left point). Horizontal dashed lines represent equal mortality rates for boys and girls.**

**eFigure 2 – Distribution of sex ratio residuals of under-five mortality**
